# Empathic Conjectures in Emotionally Focused Couple Therapy (EFCT): A Process Microanalytic Study

**DOI:** 10.1111/jmft.70075

**Published:** 2025-09-18

**Authors:** Hamed M. Fatahian‐Tehran, Simran Chatha, Robert Elliott

**Affiliations:** ^1^ Human Development and Family Studies Michigan State University East Lansing Michigan USA; ^2^ Psychological Sciences and Health University of Strathclyde Glasgow Scotland

**Keywords:** emotional deepening, emotionally focused couple therapy, empathic conjectures, interpretation, therapeutic alliance, therapist response modes

## Abstract

Empathic conjectures are a key therapist response mode in Emotion(ally)‐Focused Therapy, used to deepen emotions and strengthen attachment bonds; however, there is little research on them. This process microanalytic qualitative study analyzed 10 sessions, publicly available example of Emotionally Focused Couple Therapy (EFCT). We identified two core features: (1) guessing, where therapists articulate clients' implicit experiences, and (2) meaning match, linking conjectures directly to observable or inferable client experiences. Using a four‐point confidence rating scale, we found that 88.6% of high‐confidence conjectures were delivered tentatively, supporting tentativeness as important for minimizing therapeutic ruptures. Additionally, seven types of empathic conjectures were identified: feeling, relational process, narrative, formulation, reframing, evocative, and nonverbal feeling conjectures. These findings refine the definition of empathic conjectures, highlight therapist attunement, and provide valuable clinical insights for EFCT therapists. Future research should further explore empathic conjectures' impact on client outcomes and therapeutic alliance.

Emotionally Focused Couple Therapy (EFCT) is a well‐established and evidence‐based approach to couples therapy (Johnson 2019b). Initially developed by Johnson and Greenberg in the 1980s (Johnson and Greenberg 1985), emotionally focused approaches to working with couples are deeply rooted in both emotion and attachment theory, which underscore the crucial role of emotional vulnerabilities, bonds, and attachment in human relationships (Bowlby 1969). Recent research continues to demonstrate the high effectiveness of EFCT, with most recent studies indicating that approximately 70%–75% of couples move from distress to recovery, and around 90% show significant improvements in their relationships (Spengler et al. 2024; Beasley and Ager 2019; Wiebe et al. 2017).

Central to the success of these approaches is the therapist's ability to facilitate empathic connections with and between partners, which aids the creation of a strong working alliance and enables the partners to better understand and respond to each other's emotional experiences (Johnson 2019b). A core principle of EFCT is helping both partners access and express underlying adaptive feelings and unmet needs to foster the emotional connection in the relationship. This means that an important therapeutic process is to help both partners break the negative interactional patterns and reach out to “speak the unspoken.” Speaking the unspoken often involves the therapist making guesses based on their empathic resonance with the client's unspoken emotions and attachment needs (Johnson 2019b). The aim of these empathic conjectures is to take the client's experience one step forward by tentatively guessing the missing pieces, to help clarify and facilitate experiencing so new meanings can emerge (Johnson 2019b). When couples are in conflict, they often find it difficult to express vulnerable attachment and identity needs. Therefore, empathic conjectures are a key therapist response mode in facilitating emotional deepening of each client's experience and fostering emotional connection between partners in sessions.

Although the terms “interpretation” and “empathic conjecture” are used interchangeably in Emotion(ally)‐Focused Therapy (EFT) literature, in fact, empathic conjectures are not used to give clients cognitive labels or new information about themselves. Greenberg and Elliott (1997) have compared empathic and interpretive responses on the dimensions of aim, function, degree of inference, target, and the created role relationship. They defined empathic conjectures as tentative guesses at implicit client experiences from a shared frame of reference. These conjectures capture an unstated significant aspect of the client's current experience, thereby facilitating deepening and acknowledgment of that experience. This study seeks to delve into the intricate dynamics of empathic conjectures in the EFCT process.

Depth of interpretation in psychotherapy involves the therapist's ability to uncover and address the unconscious (or unspoken) emotions and motivations of the client, aiming to reach the core psychological factors contributing to a client's challenges (Gabbard 2004). Speisman's (1959) study on varying levels of interpretation found that moderate interpretations, which closely resemble empathic conjectures, resulted in the least resistance. These “interpretations” involve the therapist connecting different aspects of content, reformulating behaviors, and guessing the client's feelings by linking nonverbal expressions to emotions. Subsequent writings (e.g., Greenberg and Elliott 1997) further refined the concept of empathic conjectures, emphasizing the therapist's role in tentatively clarifying the client's experience without imposing outside interpretations.

This evolving understanding aligns with Johnson (2019a) definition of empathic conjecture in EFCT, which involves the therapist offering respectful, tentative, and emotionally attuned inferences to help clients deepen and clarify their emotional experience in the moment. Empathic conjecture in EFCT has been discussed and refined over the years, with a growing emphasis on its critical role in going beyond traditional empathy (Johnson 2019b) in enhancing clients' sense of being heard, understood, and fostering emotional understanding and connection. Empathic conjectures require EFCT therapists to not only understand but also articulate the unspoken emotions and needs of each partner. This practice fosters a profound emotional attunement between the therapist and the couple, enabling partners to connect on a deeper level (Johnson 2019b). Prior descriptions of the use of empathic conjecture within EFCT have laid a foundation for the present study. Johnson and Greenberg (1988) discussed the significance of empathy in couples therapy, highlighting the pivotal role it plays in fostering emotional connection. Furthermore, the work of Dalgleish et al. (2016) emphasized the importance of therapist–client emotional attunement in the context of couples therapy, shedding light on the potential impact of empathic conjecture. These previous sources provide valuable insights into the therapeutic significance of empathic conjecture and its potential effects on improving emotional bonds within couples undergoing therapy.

Therefore, the goal of this study was to identify the essential features that make an empathic conjecture and to build a research‐based definition of the empathic conjecture therapist response. Building on this study‐based definition, this study also seeks to identify different varieties/types of empathic conjectures.

## 1 Method

This process microanalytic study was guided primarily by a generic descriptive–interpretive qualitative research (GDI‐QR) approach (Elliott and Timulak 2021). GDI‐QR is located within the family of qualitative methods like grounded theory, consensual qualitative research, empirical phenomenology and hermeneutic–interpretive research. The descriptive aspect involves detailing what is observed in a straightforward, yet rich and nuanced manner (Elliott and Timulak 2021). The interpretive aspect goes a step further by exploring the meanings and implications of these observations, drawing insights that are informed by the researcher's understanding of the broader context and literature (Elliott and Timulak 2021). We used methods adapted from prototype theory and empirical phenomenology to identify a collection of empathic conjectures from 10 therapy sessions with the same couple to examine their meaning structure.

More specifically, we carried out a process microanalytic study, that is, a fine‐grained, moment‐by‐moment analysis of interactional sequences in therapy, allowing researchers to examine the specific mechanisms through which change occurs (Rice and Greenberg 1984). This approach often employs video recordings, transcriptions, and coding systems to capture therapist–client exchanges at a detailed level, providing insights into key therapeutic processes such as emotional shifts, alliance‐building, and intervention effectiveness (Elliott 1989). By focusing on microlevel therapeutic interactions, process microanalysis contributes to a deeper understanding of how specific therapeutic techniques function within the session, informing both clinical practice and training (Timulak 2008). At the same time, we also incorporated focused use of quantitative methods, such as response mode codes and confidence ratings, in a subordinate role to identify the responses to be described qualitatively.

### 1.1 Participants

The sessions analyzed are publicly available through Psychotherapy.net, encompassing 10 sessions with a single couple. We selected this video because it represents a consistent process of EFCT, including all three stages of the model (Stage 1, De‐escalation; Stage 2, Restructuring interactions; Stage 3, Consolidation and integration). This continuity provided a comprehensive view of the therapeutic journey and allowed us to examine the use of empathic conjectures and therapist interventions across all stages of EFCT with the same couple. Additionally, the recording is publicly accessible, and there is explicit and broad permission to use the video for training and research purposes, ensuring ethical compliance and accessibility for scholarly analysis and replication.

### 1.2 Clients

The couple sessions analyzed in this study feature Sandra and Carl, a retired white couple who had been married for 2 years when they started the couple therapy process. The couple was motivated to improve their relationship and strengthen their emotional bond, making them ideal candidates for this study. For confidentiality purposes, further identifying details about Sandra and Carl are not disclosed.

### 1.3 Therapist

The therapy sessions were conducted by an experienced therapist who is also a certified supervisor and trainer in EFCT. With over 25 years of clinical experience, the therapist brought extensive expertise in applying and teaching EFCT principles, ensuring that the interventions observed in this study are representative of high‐standard practice in this therapeutic approach.

### 1.4 Researchers

The research team included the first and second authors, who each analyzed five different sessions. The first author is a licensed marriage and family therapist and a certified EFCT therapist with extensive experience in delivering EFCT with couples. The second author is a licensed clinical psychologist and a certified emotion‐focused therapist. Both authors were learning qualitative research and are pursuing PhDs in fields related to couple and family therapy and psychotherapy. The third author supervised their work by reviewing the data biweekly and providing feedback to ensure accuracy in data analysis. As a senior certified EFT therapist and trainer, researcher in counseling and cofounder of EFT, they brought extensive experience in qualitative research and EFT. The first author is trained in both Emotionally Focused Couple Therapy and Emotion‐Focused Therapy, bringing a dual theoretical perspective to analysis and interpretation. The second and third authors approached the analysis and interpretation of data from an EFT framework. All authors were influenced by the therapist response modes in EFT literature, which informed their data preparation and analysis.

Each researcher brought unique expectations to the study based on their perspectives and expertise. The first author anticipated that the analysis would reveal additional inclusion criteria, such as identifying what distinguishes an empathic conjecture from a nonempathic response during the intervention. The second author anticipated that guessing and tentativeness would be essential aspects of empathic conjectures and was curious about studying the guessing process. These differing expectations provided a rich framework for interpreting the findings and examining the intervention's nuances.

### 1.5 Procedure

#### 1.5.1 Data Selection

The first two authors collected statements that were identified as potential empathic conjectures after watching each session on the selected video on the Psychotherapy.net website. They then recorded these therapist responses on a shared Excel sheet, which was organized into six columns: the first column for coding the responses from both the therapist and the clients, the second for session and the minute of the response, the third for a transcript of the response from the provided by the website, the fourth for identifying the type of therapist response mode (whether conjecture or another type of empathic response), the fifth for specifying the kind of empathic conjecture (if any, with types developed by the authors), and the sixth for a rating of the researchers' degree of confidence that the response was an empathic conjecture; was used to select empathic conjectures from the 10 couples therapy sessions, to examine its meaning structure. The use of a confidence rating scale assumes that an empirical definition of empathic conjectures can be identified by examining their underlying meaning structure, that is, the essential defining features. This approach also recognizes that human communication is inherently ambiguous and that response modes may overlap. By rating empathic conjectures as clear, fuzzy, unlikely, or not, the authors created room to explore both the essential and nonessential features of these responses.

The first two authors split the work on the videos, entering data into the table and auditing each other's work, particularly focusing on the four‐point scale and types of responses. They then consulted with the third author biweekly to discuss the collected data and incorporate their feedback. These two authors organized the therapist's empathic conjectures into a table, scaled each response based on the four‐point scale, and added ID codes for each statement. After collecting the data, they used a process description method of analysis. Process description is a mode of understanding and translating a participant's communications by characterizing the nature of their observable speech acts or their manner or style (Elliott and Timulak 2021). Both the first and second authors started analyzing all the empathic conjectures rated 3 (definite empathic conjecture) on the confidence rating scale to understand what the therapist was doing in clear instances of empathic conjectures. They then created an initial draft of inclusion criteria based on the microanalytic process description for statements rated 3 (indicating high confidence/full conjecture). After that, they reviewed the responses rated 1 or 2 (i.e., probably not conjecture or probably conjecture) to eliminate some criteria and find only the essential features that make up an empathic conjecture.

#### 1.5.2 Data Analysis

GDI‐QR analysis (Elliott and Timulak 2021) was used in this study to identify recurring themes and patterns related to the use of empathic conjecture in the context of EFCT. The specific procedures used in this study involved several steps to ensure a comprehensive understanding of the data.

First, data from therapy sessions were transcribed and systematically analyzed using an Excel sheet to manage and organize the information. Codes were assigned to segments of text that were deemed relevant to empathic conjecture. This initial categorization was aimed at capturing all potential instances of empathic conjecture within the sessions.

Next, the coded data were grouped into broader categories to develop themes. Each code was reviewed to identify patterns and connections, and these patterns were used to form thematic categories. The themes were then interpreted to understand their significance and relevance within the therapeutic context.

#### 1.5.3 Measure: Four‐Point Empathic Conjecture Confidence Rating Scale

For the assessment of empathic conjecture, the main researchers used the following anchor points: 0 = no conjecture, 1 = probably not conjecture, 2 = probably conjecture, and 3 = definite conjecture. The criteria for each level were clearly defined: 0 (no conjecture): Statements that clearly did not involve empathic conjecture (e.g., the therapist is delivering a response that does not contain any elements of empathic conjecture); (1) (probably not conjecture): Statements suggesting some level of empathic conjecture but lacking clear unspoken content (e.g., the therapist's response is empathic and may appear as new information, but it does not contain any other elements of conjecture); (2) (probably conjecture): Statements demonstrating a reasonable level of empathic conjecture with identifiable conjecture elements (e.g., the therapist's response may seem like a guess and be empathic, but it does not closely align with the client's previously spoken experience); (3) (definite conjecture): Statements that clearly and explicitly demonstrated empathic conjecture (In this scale, elements of guesswork and new information derived from the client's experience are present, clearly indicating empathic conjecture). Additionally, the analysis distinguished between various types of empathic responses and different categories of empathic conjectures to capture their diversity. The final themes reflected a nuanced understanding of how empathic conjecture functioned in EFCT, providing insights into its application and effectiveness in therapeutic interactions.

### 1.6 Trustworthiness

To ensure the reliability and validity of the findings, several strategies were employed. First, data were systematically coded using a detailed coding framework derived from existing EFCT literature. This approach aligns with Krefting (1991) recommendations for ensuring rigor in qualitative research, which emphasize the importance of a systematic approach to coding and analysis. Additionally, the coding process involved iterative discussions and revisions among the first two authors, and occasionally the third author, to refine the themes and subthemes that emerged. To further enhance the credibility of the analysis, multiple validation strategies were applied by comparing the findings between the first two authors with existing EFCT research and through regular consultations with the third author, an expert supervisor and trainer in EFT. These steps collectively contribute to the rigor of the study, addressing common critiques associated with qualitative research as outlined by Krefting (1991) and ensuring that the findings were robust and reliable.

## 2 Results

As Table 1 indicates, we identified 123 definite and 56 likely empathic conjectures. Anecdotally, we can say that these were found across all three phases of the treatment.

**Table 1:** Number of empathic conjectures on the four‐point confidence rating scale and number of tentative responses.

Four‐point confidence rating scale	Number of empathic conjectures	Tentative responses	Nontentative responses
3 Definite empathic conjectures	123	109 (88.6%)	14 (11.3%)
2 Probable empathic conjectures	56	34 (60.7%)	22 (39.2%)
1 Unlikely to be empathic conjectures	7	3 (42.8%)	4 (57.1%)
0 Not empathic conjecture	0		

### 2.1 Defining Criteria for Empathic Conjectures

The study aimed to find the essential features that make an empathic conjecture and built a research‐based definition of empathic conjectures as a therapist response mode. After identifying the common features across all 123 empathic conjectures as definite empathic conjectures, we identified two main inclusion criteria for empathic conjecture:

#### 2.1.1 Inclusion Criterion 1: Guessing

The first criterion agreed upon by the authors was guessing or inference, which refers to the therapist sensing into the client's implicit experience and articulating something that has not been said explicitly, to seek confirmation or clarification. The process of guessing was often indicated by signs of tentativeness, such as, “maybe,” “I guess,” “I think,” “like…,” and “so….” Here is an example to illustrate this criterion (sign of tentativeness in bold):

Sandra (C8.1/Session 4): If I didn't care about him, let him get skin cancer. Screw it. I don't give a damn. Let him die.

Therapist (T8): Right. **It's like,** I'm pointing those things out to him because he matters to me, because I want us to be together. I want us to work out.

The therapist, here, is guessing that Carl matters to Sandra and she wants their relationship to work out from Sandra's response of “if I didn't care….” This guess is the therapist's attempt to speak the unspoken and feel into Sandra's anger to explore what lies underneath it.

#### 2.1.2 Inclusion Criterion 2: Meaning Match

When we identified the criterion of guessing, we also noticed that not all guesses were empathic conjectures; instead, the guesses varied in how close they remained to the client's immediate experience. Therefore, the second criterion was described as “meaning match”; that is, it was generally possible to identify elements in the client's previous expression that the therapist could plausibly have used to guess something that the client had not yet spoken out loud. A meaning match often included elements from the client's verbal or nonverbal expression, but sometimes it appeared to come from the therapist's bodily felt sense or resonance with the client's experience. Here is an example of meaning match from the first session (meaning match indicators in bold):

Carl (C15.1/Session 1): I understand. I don't know what I do with it. I just try to work through it as much as I can.

Therapist (T15.2/Session 1): And you try to work through it. You kind of try to think it through, problem‐solve it in some way?

Carl: Yeap!

Therapist: Yeah, **you look** very sad **right now**, Carl. Is that sadness **on your face**‐.

Here, the therapist notes Carl's nonverbal expression of sadness, which provides the basis or a meaning match to guess that Carl is feeling sad in the moment. This enables the therapist to approach the unspoken sadness.

Guessing and meaning matching are closely interconnected. In both examples given so far, a meaning match was evident in the client's verbal or nonverbal response before the therapist's guess. This observation raised an intriguing question: how far back in the client's response can the therapist draw from to identify a meaning match and make a guess that is close to the client's experience? Importantly, the analysis highlighted the potential for the therapist's guess to extend beyond the client's frame of reference, if the meaning match is too distant. Additionally, it was noted that an empathic refocusing response (where the therapist offers the client an opportunity to return to an uncomfortable experience they introduced and then moved away from) might precede an empathic conjecture when the meaning match spans several speaking turns.

#### 2.1.3 Exclusion Criterion—Interpretation

We also explored the therapist's ability to identify a meaning match and the broad range of unspokenness that could occur. This led us to develop a scale to categorize the degree of unspokenness and to identify interpretation as an exclusion criterion for empathic conjecture:

- 0: No guessing occurs.
- 1: The guess is immediately identifiable to an observer seeking a meaning match, such as building on an empathic formulation (= weak conjecture, merging into empathic reflection).
- 2: The guess is not immediately apparent but can be identified by the observer when they search for a meaning match.
- 3: The meaning match may not be explicitly identifiable, and the guess is instead based on the therapist's empathic resonance with the client's implicit experience (= resonance conjecture).
- 4: There is no discernible meaning match, and the therapist attempts to share an insight about the client that the client may not yet be aware of (= interpretation).

We used this scale to rate all the empathic conjectures and identified an exclusion criterion for interpretation (responses rated 4 on this scale). We defined interpretation as the therapist sharing their understanding of the client's experience with the intention of telling them something they did not already know about themselves. This sort of guess thus comes from the therapist's frame of reference, with the intention to deliver “news” or “insight” to the client about their experience. The results (shown in Table 2) indicated that across 10 sessions, there were a total of 2 responses rated as 4 on this scale that fit our definition of interpretation. This example is from session 7:

Sandra: We're going to go up and see if Carl can sail a boat. Well, a little bit anyway.

Therapist: Right, she is going to share some of her skill with you.

Sandra: No, I'm going to be a bystander. I'm going to be a passenger.

Therapist: You're not going to be the teacher.

Sandra (C1.2/Session 7): Right, I'm going to ssshhhh.

Therapist (T1.2): But as he learns, that's part of your going together, is being able to sail together.

**Table 2:** Empathic conjectures across a range of unspokenness.

Degree/range of unspoken	Number of empathic conjectures
1	120
2	60
3	5
4	2

As Carl talks about making plans to sail and Sandra mentions she shall be just a passenger, the therapist offers something from her own frame of reference that sailing together is a part of being together and delivers it nontentatively as insight to the clients.

### 2.2 Recommended Criterion: Tentativeness

Initially, we included tentativeness as a third inclusion criterion for full empathic conjectures. However, upon review, we found examples of empathic conjectures rated as 3 (definite conjecture) on our scale that did not demonstrate tentativeness. As a result, we reclassified it as a recommended rather than a required criterion. We defined tentativeness as a speaking style that conveys uncertainty or openness, either through vocal tone or word choice, making it clear that the therapist is offering a guess that may or may not align with the client's experience. Tentativeness can sometimes be conveyed explicitly through a “fit question,” such as “Am I following you right?” following the empathic conjecture. Alternatively, it may be communicated through a tentative vocal tone or words that convey uncertainty like “maybe,” “I guess,” “like,” and so on, as in the following example (signs of tentativeness in bold):

Carl: Well, I'm concerned, and I feel upset with myself for I didn't realize‐‐ some things I realized, yeah. I knew about the history of the medical issues, the members of the family. But—.

T18 (Session 4): You can relate to her upset, **I think is what I'm hearing**.

Here is another example of a sequence of empathic conjectures: the first is presented evocatively without tentativeness, while the second demonstrates tentativeness using phrases such as “I guess” and “maybe” (in bold):

Carl (C9.3/Session 1): Yeah, I want this to work. And I really want us to have a good marriage.

Therapist (T9.3): I want it to work and I want to have a good marriage. I want it enough that I'm trying to convince myself that this heart stuff doesn't matter.

Carl (C9.4): Right.

Therapist (T9.4): But I end up feeling stupid, **and I guess, maybe** alone and lonely, **maybe, huh?**

Out of 123 responses rated 3 on the four‐point confidence rating scale for empathic conjecture, 109 exhibited signs of tentativeness (refer to Table 1, third column). This was expressed either explicitly through fit questions or uncertainty‐related words, or implicitly through the therapist's tonal qualities, such as rising intonation or a slower pace that conveyed doubt or a sense of guessing. Similarly, 34 out of 56 responses rated 2 also displayed these tentative characteristics.

Further analysis of nontentative empathic conjectures rated 3 revealed that 11 out of 14 of these responses were preceded or followed by tentative responses, raising the possibility that tentativeness can sometimes be implied from context. Additionally, 9 out of 14 of these responses were rated 1 on the range of unspoken scale, that is, very close to what the client had expressed, raising the possibility that the therapist did not see these as conjectures.

## 3 Types of Empathic Conjecture

We also analyzed several types and frequencies of empathic conjecture used by the therapist during EFCT with the couple. The findings (as shown in Table 3) highlight a diverse range of conjectures employed to facilitate deeper emotional connection and understanding between partners.

**Table 3:** Types of empathic conjecture with examples.

Category	#	Example	Definition
Narrative conjecture	29	1‐T1: So it's really been a life skill for you to learn how to do this. But somehow in this relationship, something happens when you say‐‐I'm guessing‐‐let's go have fun, and Carl is there‐‐stuck there, or not giving it over or something. Something happens, yeah? Like you're trying to call him forth, call him out of it, something? That's a place where you guys get kind of stuck?	A conjecture that reflects an understanding of the client's narrative or story, often aimed at capturing the essence or underlying themes.
Feeling conjecture	54	1‐T2.2: So the sailboat thing made it particularly difficult? Like that was another stressor, that was another way, and him feeling bad about it is another way. Somehow you feel pressure about‐‐we're not enjoying what we ought to be enjoying.	A conjecture that identifies and reflects the emotional experiences or states expressed or implied by the client, focusing on feelings and affective responses.
Formulation conjecture	17	1‐T4: So there's not an escape now. So normally what you would do when this pressure hits or you're confronted in some way with extra pressure or you feel threatened around some way of being, like the cockroaches, or “I need to be on guard, things could be going wrong.” And it doesn't feel like maybe you guys are a team or Carl's attending to the things that would keep you safe? Is that OK? Is that kind of right?	A conjecture that summarizes or encapsulates the client's concerns, dilemmas, or presenting issues in a concise and coherent manner, often in the form of a hypothesis or conceptualization.
Reframing conjecture	18	1‐T6: You're saying when we get all this tension between us, and especially if I see you tiptoeing around me, I get really self‐conscious, and I start to feel bad like I am that monster. I'm the pink elephant. I'm somebody you don't want to be close to, maybe. And then maybe I'm more quick to criticize, or these little things spark me up more.	A conjecture that offers an alternative perspective or interpretation of the client's experience, aiming to reframe or shift the meaning or significance of a situation or problem.
Relational process conjecture	50	9‐T6: You moved out of fear just now and into some confidence, didn't you? What happened? He reassured you.	A conjecture that focuses on the dynamics or patterns of interaction between couples, highlighting relational themes or processes that emerge during the therapy process.
Nonverbal feeling conjecture	8	9‐T8: She glows. She glows affection, doesn't she? It just like beams out of her.	A conjecture that acknowledges and reflects the client's emotional expressions, gestures, or body language, even if not explicitly verbalized.
Evocative conjecture	13	4‐T5: So part of what happens here for you is when you say, I'm a problem‐solver, I want to go fix things. I want to solve them. And so I go to the sleep apnea to figure out. And then Carl, you try to encourage her to take care of herself, and when he does that, you hear like, oh, no. But he doesn't take care of himself. Am I going to be in trouble again? Or am I spending money I don't‐‐ like it kind of comes back, like am I going to be in trouble again for doing something that takes care of me. Am I tracking that?	A conjecture that seeks to evoke or stimulate deeper exploration or reflection on the part of the client, often by posing open‐ended questions or making provocative statements aimed at eliciting insight or awareness.

### 3.1 Feeling Conjecture

Consistent with the therapeutic approach of EFCT, the most frequently used type of empathic conjecture was the feeling conjecture, with 54 instances. This form of conjecture emphasized identifying and reflecting the emotional experiences of clients, thereby validating their feelings and fostering emotional attunement. “That leaves you feeling alone, right?”

### 3.2 Relational Process Conjecture

The second most common type was the relational process conjecture, occurring 50 times. These conjectures focused on the dynamics and patterns of interaction between couples, highlighting relational themes that emerge during therapy. An example of this is, “You moved out of fear just now and into some confidence, didn't you? What happened?”

### 3.3 Narrative Conjecture

There were 29 instances of narrative conjectures. These conjectures captured the essence or underlying themes of the clients' stories, helping to integrate and make sense of their experiences. For instance, “So part of what happens here for you is when you say, ‘I'm a problem‐solver, I want to go fix things.’ I want to solve them. And so I go to sleep apnea to figure it out.”

### 3.4 Formulation Conjecture

Formulation conjectures were used 17 times. These involved summarizing the clients' concerns in a coherent manner, providing a structured understanding of their issues. “That my feelings and my needs are underground. I'm disconnected from this kind of awareness. And then I may come out in a stronger way. Like it's got to be my way now.”

### 3.5 Reframing Conjecture

There were 18 instances of reframing conjectures, which offered alternative, previously unspoken perspectives or understandings of clients' experiences. These conjectures aim to shift the meaning or significance of a situation, as illustrated by, “You're saying when we get all this tension between us, and especially if I see you tiptoeing around me, I get really self‐conscious, and I start to feel bad like I am that monster.”

### 3.6 Evocative Conjecture

Evocative conjectures, which were noted 13 times, seek to stimulate deeper exploration through open‐ended questions or evocative reflections. An example is, “Am I going to be in trouble again for doing something that takes care of me. Am I tracking that?”

### 3.7 Nonverbal Feeling Conjecture

There were eight instances of nonverbal feeling conjectures, where therapists attuned to clients' emotional expressions and body language even when not explicitly verbalized, for example, “She glows. She glows affection, doesn't she? It just like beaming out of her.”

These results indicate that therapists in EFCT employ a variety of empathic conjectures, with a significant emphasis on capturing and reflecting emotional states and relational dynamics. The frequent use of feeling and relational process conjectures underscores the importance of emotional attunement and understanding in facilitating therapeutic progress.

## 4 Discussion

On the basis of our results, it is evident that empathic conjectures in EFCT are intricate and multifaceted. Empathic conjectures are not uninformed guesses; they involve a deep, nuanced understanding of the client's unspoken feelings and experiences. Both main criteria of guessing and meaning match go hand in hand. The process of meaning match helps understand the nuances of the therapist's experience in making an empathic conjecture. It appears that the therapist notices something poignant or potentially important in the client's verbal or nonverbal expression and then speaks out a guess based on the therapist's empathic resonance. To connect this to the EFT literature, therapists employ empathic conjectures to support the emotional deepening process, by listening closely and responding in a way that names what is implicit within the client's experience.

Combining the criteria we found; we have attempted to form a research‐based definition of empathic conjectures. Empathic conjectures are response modes in which the therapist infers or guesses the client's implicit experience by identifying poignancy in a recent client expression or based on the therapist's bodily empathic resonance with the client's experience. It is generally recommended that these responses be offered tentatively, to allow the client opportunity to confirm or disconfirm the guess. They are not guesses from the therapist's frame of reference intended to provide the client “insight” into their own experience.

Additionally, we recognized the intricacies of the unspoken guess, leaving us with two questions: First, how far back can a therapist go in the conversation to form an empathic conjecture (the “conjecture space”). Second, what is the potential range or degree of the unspokenness; that is, how far from the client's spoken or implicit experience can a therapist response go before it becomes an interpretation?

When a therapist references something the client mentioned earlier in the conversation, several speaking turns ago, they may be introducing new content into the current moment, but are not guessing something implicit in the client's experience. In such cases, this response is considered an empathic refocusing rather than an empathic conjecture (Elliott et al. 2025). This is because the newly reintroduced content does not stem from the therapist's active process of deeply resonating with the client's current experience to make a guess. Instead, it involves revisiting a past part of the conversation to guide the client back to an uncomfortable or significant experience.

When a therapist makes a guess exclusively based on their own frame of reference by drawing on their own connections to what the client has said, the response may still be a guess, but will not involve a meaning match. This type of response does not qualify as an empathic conjecture, as it lacks the foundation of empathic resonance and is not close to the client's current experience. If the guess conveys to the client something the therapist thinks the client is unaware of about themselves, it shifts from being an empathic conjecture to an interpretive conjecture.

Our analysis also revealed that 88.6% of empathic conjectures rated 3 on the confidence rating scale were delivered tentatively. It makes sense for the therapist to show some uncertainty while guessing, because it leaves room for the client to confirm or refute the guess. While empathic conjectures can support the emotional deepening process, they also carry a level of risk of therapeutic rupture if the client does not agree with the guess (Elliott et al. 2023). For this reason, we included tentativeness as a recommended criterion for delivering empathic conjectures effectively. It was fascinating to observe sequences of alternate empathic conjectures delivered tentatively and nontentatively. For example, a therapist might offer a series of empathic conjectures, each building on the previous one, if there was strong agreement from the clients. Alternating between tentative and nontentative empathic conjectures could develop a sense of contextual tentativeness between the therapist and client. This highlights the need for further exploration of therapist practices in balancing their epistemic authority (i.e., about the client's experience) while delivering empathic conjectures, as in Smoliak et al. (2022) study on process‐guiding responses. Additionally, we found that 9 out of 14 nontentative responses were not risky guesses since they stayed fairly close to the client's reported experience, which further points to the importance of studying how therapists navigate the balance between not claiming too much epistemic authority and empathically facilitating emotional deepening.

Another interesting finding is that more than half of the empathic conjectures were made after empathic formulation responses. This could indicate that this EFCT therapist at least, typically first formulated the couple's cycle, before attempting to deepen their experience by following it up with empathic conjectures.

## 5 Clinical Implications

A key implication for EFT or other therapies is that empathic conjectures are not mere wild or intellectual guesses but are deeply rooted in the therapist's attunement to the client's implicit experience. Rather than relying on intellectual assumptions, therapists are encouraged to use their bodily empathic resonance to identify and articulate what feels poignant in the client's expression. Delivering empathic conjectures tentatively allows clients the opportunity to confirm, disconfirm, or elaborate on the guess. Observing moments of poignancy in the client's tone, body language, and expressions can guide the therapist in formulating empathic conjectures close to the client's experience. A key clinical implication for therapists and supervisors is the need to sharpen perceptual skills, particularly in recognizing moments of poignancy in client's expression, the therapist's meaning‐matching process and the degree of unspoken meaning in their empathic conjectures. Strengthening this awareness can also help therapists notice moments when their empathy may have missed the mark or did not resonate with the client. In addition to illuminating the nuanced process of speaking the unspoken in empathic conjectures, our findings break down the therapist's empathic process in ways that we hope will support the development of these skills.

In the context of EFT couple therapy, certain types of empathic conjecture stood out as uniquely suited to relational work, reflecting the nuanced dynamics between partners. Relational Process Conjectures are particularly distinctive, as they emphasize the patterns of interaction and emotional exchanges within the couple, shedding light on how partners influence and respond to each other in the therapy room. These conjectures are integral to identifying shifts in relational dynamics, such as moments of reassurance or conflict resolution, that are central to fostering emotional connection. Similarly, Nonverbal Feeling Conjectures capture the unspoken emotional cues expressed through body language or gestures, which are often more telling than verbal communication in intimate relationships. By recognizing and reflecting these nonverbal signals, therapists can deepen the couple's understanding of each other's emotional worlds. Together, these conjectures highlight the importance of attuning to both explicit and implicit relational dynamics, making them essential tools in the therapeutic process for couples.

In practice, the study has reinforced the importance of using empathic conjectures to deepen emotional connections and understanding between clients. It highlights the need for therapists to develop and refine their empathic responses, particularly in EFCT, where emotional attunement is critical. Learning to employ different types of empathic conjectures effectively can help therapists facilitate more profound emotional experiences and foster stronger, more resilient relationships between partners.

Future research could build on this definitional study to understand the role of empathic conjectures and their impact on the therapy process in EFCT. We also hope that this definitional study framework informed by prototype theory and empirical phenomenology can be employed for definitional research to deepen our understanding of other complex therapeutic processes across diverse clinical contexts.

## 6 Limitations

Several limitations should be recognized in this study. First, the findings may be context‐specific and may not be directly applicable to all therapeutic settings or populations, or even to other EFCT therapists. The study's qualitative nature and focus on process analysis might also restrict its ability to establish causal relationships or generalize the results. Additionally, external factors and individual differences in clients and therapists that could impact the effectiveness of EFCT may not be fully accounted for in this study. Specifically, this study was conducted with only one couple and analyzed the responses of a single therapist, which limits the generalizability of the findings. The data was drawn from training videos by an expert trainer rather than routine therapy sessions conducted by trainee or other experienced therapists. While these videos provide rich examples for analysis, they may not fully capture these processes as they occur in real‐world therapy practice with trainee and experienced therapists. The focus on a specific therapeutic setting, couple therapy, also means that the results may not apply to other contexts or types of therapy. Future research should seek to include a larger and more diverse sample of couples including those from racial, sexual, and gender minority backgrounds as well as a wider range of therapists to validate the present findings and further investigate the use and impact of empathic conjectures across diverse therapeutic settings. On the other hand, we believe that we have demonstrated the value of the process microanalytic method used here for illuminating the use of an important form of therapist response. We recommend further research on empathic conjectures, examining their immediate and delayed effects on clients and identifying the determinants of effective and ineffective empathic conjectures.
